# Clinical drug trials in general practice: a 10-year overview of protocols

**DOI:** 10.1186/1745-6215-14-162

**Published:** 2013-06-01

**Authors:** Anja Maria Brænd, Kaspar Buus Jensen, Atle Klovning, Jørund Straand

**Affiliations:** 1 Department of General Practice, Institute of Health and Society, Faculty of Medicine, University of Oslo, PO Box 1130, Blindern, Oslo N-0318, Norway

**Keywords:** Clinical trial, Drug industry, General practice, Patient selection, Randomized controlled trials

## Abstract

**Background:**

Drugs predominantly prescribed in general practice should ideally be tested in that setting; however, little is known about drug trials in general practice. Our aim was to describe drug trials in Norwegian general practice over the period of a decade.

**Methods:**

The present work concerns a 10-year retrospective study of protocols submitted to the Norwegian national medicines agency (1998 to 2007) identifying all studies involving general practitioners (GPs) as clinical investigator(s). We analyzed the number of trials, drug company involvement, patients, participating doctors, payment, medications tested and main diagnostic criteria for inclusion. We also analyzed one trial in greater detail.

**Results:**

Out of 2,054 clinical drug trials, 196 (9.5%) were undertaken in general practice; 93% were multinational, 96% were industry funded and 77% included patients both from general practice and specialist care. The trials were planned to be completed in the period 1998 to 2012. A total of 23,000 patients in Norway and 340,000 patients internationally were planned to be included in the 196 trials. A median of 5 GPs participated in each trial (range 1 to 402). Only 0.7% of 831 GP investigators had general practice university affiliations. Median payment for participating investigators was €1,900 (range €0 to 13,500) per patient completing the trial. A total of 30 pharmaceutical companies were involved. The drugs most commonly studied were antidiabetics (21%), obstructive airway disease medications (12%), agents acting on the renin-angiotensin system (10%), and lipid modifying agents (10%). One trial, presented in more detail, had several characteristics of a seeding or marketing trial.

**Conclusions:**

Only one in four drug trials involving general practice were solely general practice trials and almost all were industry initiated without input from academic general practice. There was a large variation in the number of patients, participating doctors, and economic compensation for trial investigators, with some investigators receiving substantial payments.

## Background

About 90% of all drug prescriptions for outpatients are issued in general practice [1]. Patients in general practice are more likely to have less severe disease, and more undifferentiated symptoms than selected patients in secondary or tertiary health care, however, multimorbidity is common [2]. If a study population in a drug trial differs significantly from the population where the drug is most likely to be prescribed, the external validity of the trial may be impaired [3]. To avoid this uncertainty, many have argued that more clinical drug trials should be conducted in primary care settings to ensure that the benefits are proportionate to the risks and costs of the treatment for patients in general practice [4,5]. Clinical drug trials may serve different purposes, from research of effectiveness, to marketing [6]. Trials with the main purpose of introducing new drugs to prescribers are often referred to as ‘seeding trials’. Various criteria for seeding trials have been proposed (Table 1) [7-9], and it is characteristic that the trials are designed to make many clinicians familiar with a new drug. Being frequent prescribers, general practitioners (GPs) are probably of particular interest for designers of seeding trials; this is also because GPs are relatively independent in their decision-making processes.

**Table 1:** **Case study: the ‘On-demand Nexium Evaluation’ (ONE) trial**^**a**^

**Key characteristics of seeding trials**[7-9]	**Does this apply for the ONE trial?**
Tests a new drug recently or about to be licensed	Yes: application year 2000, study completed by the end of 2001; esomeprazole licensed in 2001
Many well established competing products	Yes: omeprazole from 1989, lansoprazole and pantoprazole from 1995
Many patients included	Yes: 2,500 patients (the trial with most Norwegian patients to be included). In addition, similar studies have been conducted in other countries (see main text).
Frequent prescribers in the role of clinical investigators	Yes: 402 general practitioners (GPs; the study involving most Norwegian GP investigators)
Often redundant as they are not designed for answering a scientific problem	Probably yes
Unreasonably high payments for the investigators	No: €750 which is below the 25th percentile for all trials
Results are often not published	No: there were three publications
Conducted by drug company marketing departments	Unknown

^a^Full trial title: ‘Long-term treatment of patients with symptomatic gastroesophageal reflux disease (GERD) comparing costs and efficacy over six months of treatment with esomeprazole 20 mg q.d on demand treatment or esomeprazole 20 mg q.d continuous treatment or ranitidine 150 mg b.i.d. continuous treatment. An open, randomised, multicenter study. On-demand Nexium Evaluation - ONE’. Sponsor: AstraZeneca.

In the UK between 1984 and 1989, general practice trials initiated by pharmaceutical companies were judged to have a low output of clinically relevant results [10]. The concerns addressed in that audit were mainly payments to GPs, information and safety issues, shortfall of investigators and patients, leading to inconclusive results, and low publication rates [10]. Among general practice studies published 1991 to 1996 in the *BMJ*, the *British Journal of General Practice*, and *Family Practice*, only 6% were randomized controlled trials, which was pointed out as a major challenge for the general practice research community [11]. Some Norwegian data regarding clinical drug trials have previously been reported, based on research applications submitted to the Research Ethical Committees (REC) [12-14] and the Norwegian Medicines Agency (NoMA) [15,16]. Between 68% and 85% of the trials were initiated by pharmaceutical industry [13-15], however, neither of these reports highlighted studies in general practice.

From the 1970s, a subcommittee of the Norwegian College of General Practitioners had a voluntary agreement with the pharmaceutical industry to assess protocols for studies to be performed in general practice [17,18]. If the committee judged the trial to be clinically relevant and of sound scientific quality, GPs were recommended to participate in the trial. However, by the turn of the millennium, the committee voiced critical comments regarding some trials judged to be marketing (due to features of seeding trials) rather than research [17]. Since then, this voluntary quality and relevance check has been largely bypassed by the industry [19], and information regarding industry initiated research in Norwegian general practice has thus become an almost hidden reality except for the investigators themselves. This prompted the Norwegian College of General Practitioners in 2008 to fund research to gain systematic knowledge of the drug research carried out by GPs. This included establishing an overview of all trials conducted, exploring whether GPs were subject for marketing trials, and assessing the relevance of the research questions addressed, as well as the studies’ validity and publication output. The College requested this knowledge due to possible implications for their policy for future professional development within the discipline of general practice.

The aim of the present study was to establish a descriptive basis regarding clinical drug trials conducted in general practice by research areas and trial characteristics. As most clinical drug trials are multinational, this study also should concern research in general practice outside of Norway.

## Methods

In Norway, the only complete national archive for drug trials is held by NoMA. Based on a hand search of the NoMA archive for the 10-year period 1998 to 2007, we describe all clinical trials planned to be carried out in general practice by their funding, clinical setting, drugs, diagnoses, patients, clinical investigators and economic compensations for the investigators. More detail will be given regarding one of the trials in year 2000, which the Norwegian College of GPs discouraged GPs to join [17], to give the reader a better sense of what we consider a seeding trial. Ethical approval for our study was not required.

### Dataset

In Norway, all clinical pharmaceutical trials regardless of setting need approval from NoMA, the national, regulatory authority for new and existing medicines. Clinical trials are regulated by international and national laws, in which there were amendments between 1998 and 2007, and from 2004 the European Directive 2001/20/EC has been implemented in the Norwegian regulation [16]. By hand searching NoMA’s total paper archive, protocol by protocol, we were able to identify application forms, protocols and other correspondence received by NoMA regarding applications for clinical drug trials between 1998 and 2007, the 10-year period before conversion to electronic registration. The computed total number of trial applications during this decade was 2,054 [16,20] (personal communication from I. Aaløkken, NoMA). The main hand search was performed in May to August 2008 by KBJ. Supplementary data collection and verification was performed by AMB between November to December 2011 for a total of 89 studies. No formal inter-rater reliability calculations were performed, but no significant errors or discrepancies were disclosed. A random check of non-included studies in the archive did not reveal any additional general practice trials. Applications for trials planned to take place solely or partly in general practice were included. General practice trials were defined as trials where the address and/or titles indicated that at least one of the Norwegian clinical investigators worked in general practice.

### Variables: trial characteristics

For included studies, we recorded study title, medication tested in terms of its Anatomical Therapeutic Chemical (ATC) classification code [21], funders, whether the study was planned exclusively to be conducted in general practice or also in specialist care settings, whether it was a national or international study, and trial phase based on explicit information in the application ((I) study of toxicity and side effects; (II) study of dose-response; (III) comparison with established treatment or placebo; or (IV) post-marketing study, gaining broader experience) [6]. We defined a study as industry initiated when a pharmaceutical company funded the study, wrote the study protocol or conducted the study, either the company itself or via a contract research organization. If the study drugs were provided free of charge, but the trial was otherwise designed and conducted independently under the responsibility of a GP researcher, it was defined as researcher initiated. There were no trials with unclear funding. We recorded the number of patients planned to be included internationally, and at Norwegian trial sites, the number of clinical investigators involved, trial duration, and if available, economic compensation for physicians involved. We also identified clinical investigators who were GP academics, that is, having affiliations to Norwegian general practice university departments. The main diagnostic criteria for inclusion in a study were based on the study title, and when necessary supplemented with information from the protocol and categorized according to the *International Classification of Primary Care*, second edition (ICPC-2) [22]. One of the authors (AMB) performed the categorization, and when in doubt, consensus was reached through discussions between AK, JS and AMB.

### Data analysis

Data were compiled in a spreadsheet. To compare planned number of patients to be enrolled in trials partly or entirely to be undertaken in general practice, we used the independent samples Mann-Whitney U test, and otherwise we used descriptive statistics. Due to data skewness, median values are presented rather than means. We roughly estimated investigator incomes by multiplying median payment and median number of patients planned recruited divided by median number of investigators. Statistical analyses were performed using SPSS statistics (PASW Statistics 18; SPSS Inc., Chicago, IL, USA).

### Case study

One outlying case is described in greater detail. For this trial, additional information is given on patients, interventions, comparison and outcome based on the study protocol. A systematic literature search was performed to identify publications arising from the trial (date of literature search, 17 September 2012). The trial was assessed according to the proposed criteria for seeding trials outlined in Table 1[7-9].

## Results

### Dataset: general practice drug trials

**Figure 1:**
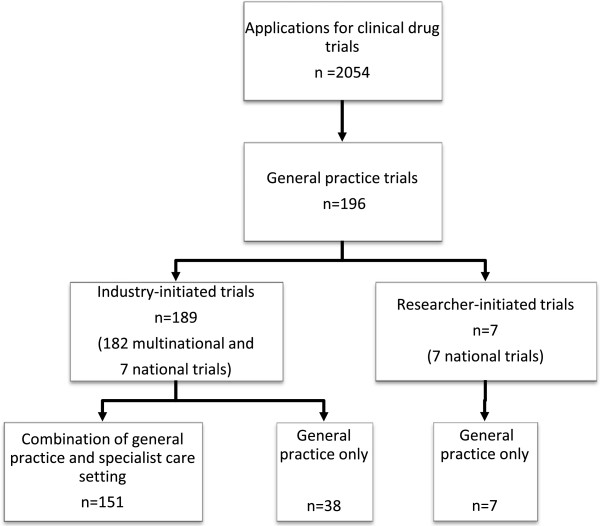
**General practice clinical drug trials in Norway 1998 to 2007.** Applications to the Norwegian Medicines Agency (NoMA) for industry initiated or researcher initiated clinical drug trials conducted entirely or partly in general practice as identified by hand search of the clinical trials archive. A total number of 2,054 clinical drug trials during the time period was calculated (personal communication from Ingvild Aaløkken, Head of section, Preclinical assessment and clinical trials, NoMA, 2008; see also [16,20]).

During the 10-year period of applications, a total of 196 studies (9.5% of all 2,054 clinical drug trials) were planned to be conducted entirely or partly in a general practice setting. Of these, 189 (96.4%) trials were industry initiated and 7 (3.6%) were researcher initiated (Table 2). The trials were planned to be completed in the period 1998 to 2012. Figure 1 shows that the majority of the 196 trials had trial sites both in general practice and in specialist care. The majority of the trials were multinational. Only 45 trials (2.2% of all) were planned conducted entirely in general practice. The number of participating countries per trial varied from 1 to 50 (median: 9, interquartile range 5 to 17); however, for 112 trials information regarding the number of countries was incompletely stated.

**Table 2:** Clinical drug trial applications in Norway 1998 to 2007

**Year**	**General practice trials**			**All clinical trials**		
	**Industry initiated (GP only)**	**Researcher initiated**^**a**^	**Total (GP only)**	**Industry initiated**	**Researcher initiated**	**Total**
1998	17 (7)	0	17 (7)	186	50	236^b^
1999	16 (6)	0	16 (6)	161	28	189^c^
2000	23 (10)	1	24 (11)	173	65	238^d^
2001	22 (1)	0	22 (1)	158	62	220^d^
2002	16 (5)	2	18 (7)	143	50	193^d^
2003	14 (1)	1	15 (2)	151	50	201^d^
2004	22 (2)	1	23 (3)	171	60	231^d^
2005	18 (2)	1	19 (3)	139	55	194^d^
2006	20 (1)	1	21 (2)	113	60	173^d^
2007	21 (3)	0	21 (3)	126	53	179^d^
Total	189 (38)	7	196 (45)	1,521	533	2,054
Mean/year	18.9 (3.8)	0.7	19.6 (4.5)	152.1	53.3	205.4

Applications at the Norwegian Medicines Agency (NoMA) for clinical drug trials conducted entirely (general practitioners (GPs) only) or partly in general practice identified by hand searching of all the clinical trials in the archive. For comparison, an overview of all clinical trials is included.

^a^All researcher-initiated trials were GP only.

^b^Personal communication from Ingvild Aaløkken, Head of section, Preclinical assessment and clinical trials, NoMA, 2008.

^c^See [16].

^d^See [20].

### Trial characteristics

A total of 30 different pharmaceutical companies applied for drug trials in general practice during this 10-year period. The initiators (number of trials) were GlaxoSmithKline (39), AstraZeneca (32), Novartis (21), MSD (19), Pfizer (11), Novo Nordisk (9), Boehringer Ingelheim (8), Roche (7), Lundbeck (5), and Schering-Plough (4). In addition, 5 companies initiated 3 trials, 4 companies initiated 2 trials, and 11 companies initiated 1 trial. The top 5 companies in terms of Norwegian market share [23] were responsible for 107 (55%) of all trials.

Table 3 shows the number of patients and physicians involved and estimated economic compensation for the investigators. More than 23,000 Norwegian patients were to be included in the 196 trials, and almost 340,000 patients included internationally. In 6 trials the planned number of included patients was more than 10,000, and in 10 trials the number was less than 100 patients. The recruitment targets did not differ between trials in general practice only and those undertaken in mixed health care settings (*P* = 0.91). Only 6 (0.7%) out of 831 clinical investigators were general practice academics, 3 of whom were only involved in trials without commercial sponsors. Information regarding trial investigators’ payment was missing in 90 applications, 73 of which were from the period 1998 to 2002.

**Table 3:** Characteristics for clinical drug trials in general practice

	**Patients planned for inclusion**			**Clinical investigators**		**Payment**	
	**Norway**	**All countries**	**Trial duration, weeks**	**General practice**^**a**^	**All sites in Norway**	**Investigators' payment per patient, €**^**b**^	**Estimated yearly income per investigator, €**^**c**^
Median	60	672.5	24	5	7	1,900	1,600
Minimum to maximum	8 to 2,500	8 to 31,000	1 to 288	1 to 402	1 to 402	0 to 13,500	0 to 59,000
2.5 to 97.5 percentile	13 to 560	50 to 14,368	3 to 240	1 to 37	1 to 61	0 to 7,500	0 to 25,000
Sum	23,635	337,921		1,920	2,475		

This table shows trial characteristics of applications at the Norwegian Medicines Agency (NoMA), 1998 to 2007, for 196 clinical drug trials conducted solely or partly in general practice identified by hand searching of the archive.

^a^General practitioners (GPs) participated in clinical trials from 1 to 36 times, with median 1 and interquartile range 1 to 2. A total of 831 unique GPs participated.

^b^Information missing in 90 applications.

^c^Calculated using median payment and median number of patients recruited per investigator. This payment covers all expenses connected with the trial for the investigator.

We were able to record the study phase in 122 trials, out of which none were phase I studies, 11% were phase II studies, 61% phase III and 27% phase IV studies.

Drugs from 30 different therapeutic groups were investigated in the trials (Table 4). The largest groups were antidiabetics, drugs for obstructive airway diseases, agents acting on the renin-angiotensin system, and lipid modifying agents. The top 5 therapeutic subgroups represented 121 (59%) of all drug groups tested, the top 10 represented 158 (78%). Only one of the trials investigated medication discontinuation.

**Table 4:** **Anatomical Therapeutic Chemical classification (ATC code**^**a**^**) for test drugs in clinical drug trials in general practice**

**Anatomical main ATC group**	**n**	**%**	**Therapeutic ATC subgroup**	**n**	**%**	**Rank (1 to 10)**
A: Alimentary tract and metabolism	52	25.5	A02: Drugs for acid-related disorders^b^	7	3.4	7
			A03: Drugs for functional gastrointestinal disorders	2	1.0	
			A08: Antiobesity preparations, excluding diet products	1	0.5	
			A10: Drugs used in diabetes^b^	42	20.6	1
B: Blood and blood-forming organs	4	2.0	B01: Antithrombotic agents	4	2.0	
C: Cardiovascular system	46	22.5	C02: Antihypertensives	2	1.0	
			C08: Calcium channel blockers^b^	2	1.0	
			C09: Agents acting on the renin-angiotensin system	21	10.3	3
			C10: Lipid modifying agents^b^	21	10.3	3
D: Dermatologicals	2	1.0	D01: Antifungals for dermatological use	1	0.5	
			D04: Antipruritics, including antihistamines, anesthetics, etc.	1	0.5	
G: Genitourinary system and sex hormones	8	3.9	G02: Other gynecologicals	1	0.5	
			G03: Sex hormones and modulators of the genital system	2	1.0	
			G04: Urologicals	5	2.5	10
H: Systemic hormonal preparations	3	1.5	H02: Corticosteroids for systemic use^c^	2	1.0	
			H05: Calcium homeostasis	1	0.5	
J: Anti-infectives for systemic use	22	10.8	J01: Antibacterials for systemic use^b^	4	2.0	
			J05: Antivirals for systemic use	12	5.9	6
			J07: Vaccines	6	2.9	9
L: Antineoplastic and immunomodulating agents	2	1.0	L01: Antineoplastic agents	2	1.0	
M: Musculoskeletal system	15	7.4	M01: Anti-inflammatory and antirheumatic products	13	6.4	5
			M05: Drugs for treatment of bone diseases	2	1.0	
N: Nervous system	23	11.3	N02: Analgesics	7	3.4	7
			N04: Anti-Parkinson drugs	2	1.0	
			N05: Psycholeptics^c^	5	2.5	
			N06: Psychoanaleptics^c^	5	2.5	
			N07: Other nervous system drugs	4	2.0	
R: Respiratory system	26	12.7	R01: Nasal preparations	1	0.5	
			R03: Drugs for obstructive airway diseases^c^	24	11.8	2
			R06: Antihistamines for systemic use	1	0.5	
Missing information				1	0.5	
Total number of ATC codes				204	100	

This table shows the ATC codes of drug being tested identified from applications to the Norwegian Medicines Agency (NoMA) for 196 clinical drug trials between 1998 and 2007 conducted entirely or partly in general practice through hand searching the archive.

^a^See [21].

^b^Additional ATC code in a total of eight trials with two ATC codes registered.

^c^ATC code in researcher-initiated trials; for the groups H02, N05 and R03 there were two trials.

The main diagnostic inclusion criteria represented 44 different diagnoses (Table 5), the top 5 of which made up 114 (52% of the inclusion criteria) and the top 10 made up 146 (67%). In 14 trials no diagnosis was applicable, that is, healthy people, subjects over a certain age, smokers, and patients using baby aspirin.

**Table 5:** **Main diagnostic criteria for inclusion classified in terms of *International Classification of Primary Care *(ICPC) diagnoses**^**a **^**for clinical drug trials**

**ICPC diagnosis**	**n**	**%**	**GP only**
T90: Diabetes, non-insulin dependent	44	20.2	1
K86: Hypertension, uncomplicated	22	10.1	7
R96: Asthma	19	8.7	4
T93: Lipid disorder	18	8.3	2
R80: Influenza	11	5.0	7
L89: Osteoarthrosis of hip	7	3.2	2
L90: Osteoarthrosis of knee	7	3.2	3
N89: Migraine	7	3.2	1
R95: Chronic obstructive pulmonary disease	6	2.8	2
P76: Depressive disorder	5	2.3	2
K78: Atrial fibrillation/flutter	4	1.8	
L88: Rheumatoid/seropositive arthritis	4	1.8	
T82: Obesity	4	1.8	
D07: Dyspepsia/indigestion	3	1.4	2
P70: Dementia	3	1.4	2
T99: Endocrine/metabolic/nutritional disease, other	3	1.4	
D84: Oesophagus disease	2	0.9	2
D93: Irritable bowel syndrome	2	0.9	1
L84: Back syndrome without radiating pain	2	0.9	1
L91: Osteoarthrosis, other	2	0.9	1
L95: Osteoporosis	2	0.9	
N04: Restless legs	2	0.9	
P06: Sleep disturbance	2	0.9	
R78: Acute bronchitis/bronchiolitis	2	0.9	2
U04: Incontinence, urine	2	0.9	
Y07: Impotence, not otherwise specified	2	0.9	
A23: Risk factor, not otherwise specified	1	0.5	1
A91: Abnormal result investigation, not otherwise specified	1	0.5	
D12: Constipation	1	0.5	
K76: Ischemic heart disease without angina	1	0.5	
K99: Cardiovascular disease, other	1	0.5	
L29: Symptom/complaint, musculoskeletal, other	1	0.5	
L92: Shoulder syndrome	1	0.5	1
L93: Tennis elbow	1	0.5	1
L99: Musculoskeletal disease, other	1	0.5	
R28: Limited function/disability (respiratory)	1	0.5	
R75: Sinusitis, acute/chronic	1	0.5	1
R76: Tonsillitis, acute	1	0.5	1
R81: Pneumonia	1	0.5	
R97: Allergic rhinitis	1	0.5	
S74: Dermatophytosis	1	0.5	1
T89: Diabetes, insulin dependent	1	0.5	
X11: Menopausal symptom/complaint	1	0.5	
No diagnosis applicable	14	6.4	2
Total^b^	218	100	

Table shows main inclusion criteria classified as ICPC diagnoses identified from applications at the Norwegian Medicines Agency (NoMA) for 196 clinical drug trials between 1998 to 2007 conducted entirely (general practitioner (GP) only) or partly in general practice through hand searching of the archive.

^a^See [22].

^b^A total of 22 studies had 2 or more diagnoses as inclusion criteria; 3 studies had 3 diagnoses.

### Case study

In Table 1, more detail is given for one particular trial which the Norwegian College of GPs discouraged GPs to join [17], the ‘On-demand Nexium Evaluation’ trial, with the following clinical characteristics. **Patients:** patients with symptoms suggestive of gastroesophageal reflux disease (GERD; heartburn with or without acid regurgitation) for 3 days or more were included. Only patients with effect of treatment with esomeprazole 40 mg were randomized for comparison with ranitidine. **Intervention:** the drug tested was esomeprazole 40/20 mg daily. **Comparison:** there was initially no comparison; if treatment success in the run-in period with esomeprazole 40 mg daily, comparison was esomeprazole 20 mg daily on demand or ranitidine 150 mg twice daily. **Outcomes:** difference in direct medical costs (mean per patient) was the primary outcome, secondary objectives included health care contacts, tests and procedures, hospitalizations, patient time and travel costs, early retirement, absence from work, symptom registration, quality of life, self-perceived overall treatment effect, and patient satisfaction. Among all 196 trials in Norwegian general practice during the decade, this trial was designed to include the largest number of national GP investigators and patients. In the sample size calculations a power of 95% (beta = 0.05) was used. The significance level alpha was 5%. The study had an open design, with no blinding. There was a run-in period before randomization, and only patients responding to the high dose esomeprazole treatment were randomized and included in the intention-to-treat analyses. Per protocol analyses were also used. Three journal articles presenting results from the study were identified [24-26].

## Discussion

The main findings in this study were that only about one-tenth of all clinical drug trials in Norway involved GPs as clinical investigators recruiting patients from their practices, and only 3.6% of these were non-industry trials.

We found that the proportion of researcher-initiated clinical drug trials in general practice in Norway was minute (3.6%) compared to clinical drug trials from other fields of medicine where 15% to 32% are non-commercial studies [13-15]. Conducting clinical trials in general practice poses some typical challenges, like difficulties with study logistics and patient recruitment in a large number of small trial sites [4]. The lack of researcher-initiated trials has also been pointed out in the European Research Agenda for General Practice [5]. Irrespective of their commercial interests, this research agenda underlines that drugs mainly targeting primary care should be appropriately tested in primary care settings. Furthermore, that a stable funding and formation of research networks may facilitate non-commercial randomized controlled trials in general practice [5]. Although many have pointed to the need for clinical drug trials conducted independently of the industry, there was nonetheless a decline in the number of non-commercial randomized controlled trials in the UK between 1980 and 2002 [27]. Several systematic reviews have pointed out that drug trials financed by pharmaceutical industry tend to publish results more in favor of the manufacturer’s product than non-commercial trials, and that negative studies in this respect commonly remain unpublished [28-32]. This publication bias combined with the lack of non-industry trials in general practice might therefore contribute to an evidence-biased knowledge base.

Over the last few years, there has been a decline in the number of clinical trials in several European countries [33,34]. In Norway, the largest reduction has been in phase III studies [16]. For general practice studies we were not able to confirm this trend, even if the majority of trials in our material were phase III or IV. The trials where explicit information regarding trial phase was missing, were most likely also phase III or IV. The five companies funding most general practice trials were among the seven most profitable companies, thus largely reflecting their market positions [23].

A study from 2000 found that 84% of GPs in a part of England had been involved in research, 48% mainly in clinical trials [35]. Our figures indicate that Norwegian GPs’ participation rate in clinical trials is less than half of that in the UK; the 831 GPs involved correspond to about 20% of all Norwegian GPs in 2002 [1]. Surprisingly few GPs with university affiliations had participated in clinical drug trials, although many of them work part-time as GPs and are particularly interested in clinical research. Explanations for this finding may be that they judged the clinical relevance of the industry funded trials to be low, feared conflicts of interest, or had general negative attitudes towards collaboration with pharmaceutical companies [36]. Most GPs involved in clinical trials only participated once or just a few times, and did therefore not gain large incomes for participating. However, a number of GPs were involved in quite a few trials, giving them a substantial yearly income from trial participation. A few trials also gave outstandingly high payment for the doctors involved. Most GPs in Norway are self-employed with a major part of their income from patient payment and per capita reimbursement. It is therefore reasonable that extra workload due to participation in clinical trials is compensated. But how much is a reasonable level of compensation? Payment to clinicians for participation in clinical trials varies a lot [36,37]. Andersen *et al*. report payments of US $800 per patient enrolled for a Danish industry initiated trial [37], which is approximately one-third of the median payment in our material. Raftery *et al*. point to the striking lack of transparency in guidelines for payments for involvement in research, where GPs are among the few individual clinicians where direct personal payment still is common [36]. Concerns have been voiced that high payments may create conflicts of interest and possibly lead to unethical recruitment processes [36]. This demands rigorous ethical standards from both the general practice community and the pharmaceutical industry.

We identified antidiabetic drugs and type II diabetes to be studied most frequently. Thomas *et al*. found few published articles on diabetes in UK primary care journals in the 1990s [11]. The general use of antidiabetics in Norway increased by 27% (in defined daily doses (DDDs)) from 2004 to 2008 [38]. This may both reflect increasing diabetes prevalence but also the introduction of new drugs. Andersen *et al*. found that physicians conducting a clinical trial, significantly increased the sponsor’s share of prescribed drugs for the disease compared to GPs not involved in the trials [37], and this might be important for expanding the market. The many post-marketing studies of new and expensive insulin analogues with large market potentials have recently been criticized for limited scientific value [9]. The three largest drug groups investigated in our study were among the four most consumed drugs in Norway (in terms of DDDs) [38]. These therapeutic areas were also among the six most frequently researched topics in general practice internationally between 2003 and 2008 [5]. In Norway in years 2000 and 2004 [14], and in the UK 1980 to 2002 [27], most clinical trials were in the field of cancer and antineoplastic and immunomodulating drugs. We only identified two trials in this field. This was as expected, as GPs rarely initiate antineoplastic drug prescriptions. Only one trial studied the effects of discontinuation of (unnecessary) medication use, although this is an important topic both for quality of care and general practice research [5].

### Strengths and limitations of the study

The NoMA archive is a mandatory, complete national archive of all clinical drug trials in Norway, and it is a strength that we managed to include all trial protocols, not restricted to a specific region or to trials that had been reported in scientific publications. The large proportion of multinational trials identified also increases the external validity of our findings, which therefore may be relevant for general practice in other countries. As the identification of trials was performed by hand searching, random errors may have occurred during this process and recording of results. The main hand search was performed by only one of the authors, but supplementary searches in the archive for completing information by another author did not reveal significant errors. The total number of trials (2,054 trials) for the 10-year period was summed up from numbers provided by NoMA, and the exact total number of investigated paper files was not recorded, although the whole archive was searched shelf by shelf. Some uncertainty regarding missing files in the archive is therefore possible, however, we believe the number of missing files to be negligible due to strict control and restricted access to the state run archive. We have not addressed the number of clinical trials that were never conducted, either from lack of approval from REC or NoMA, or for other reasons, but this will be a subject of future research. The patient numbers reported involve all trial sites in Norway, and we do not know the exact number of general practice patients. Concerning trial phase, the proportion of missing data was quite large. The same applies to investigator payments, which were under-reported to NoMA, especially during the first years in the time period studied.

### Case study: a seeding trial?

The intentions for conducting a seeding trial are in general hidden and they may therefore be difficult to identify. Seeding trials have usually been disclosed based on documents from litigation processes [39,40]. Without access to internal communication within the drug company, the judgment of whether a trial is designed for marketing has to be based on several aspects. The esomeprazole trial described has many features being typical for a seeding trial, with several of the key characteristics described in Table 1[7-9].

Esomeprazole was launched in 2001 in a crowded drug group. It is the most expensive of the proton pump inhibitors, and had become the tenth most-sold medicine in Norway in 2008 calculated in terms of pharmacy retail prices [38]. The esomeprazole trial described was large, and the sample size calculations were based on a higher power than conventionally used [41]. A higher power demands larger sample sizes and more patients to be enrolled into the study. No ethical considerations regarding these issues were discussed in the peer-reviewed publications arising from the trial [24-26]. The open design and the inclusion only of patients responding to high doses of esomeprazole in the intention-to-treat analyses increased the risk of bias in favor of esomeprazole. One may question the scientific need for recruiting around 400 GPs in the trial, each GP only enrolling a handful of patients with gastroesophageal reflux, a relatively common health complaint. The large number of participating doctors was not explained or justified in the protocol or in publications arising from the trial [24-26]. Nevertheless, the trial was approved by both the regional ethics committee and NoMA without any major remarks. The general practice research committee criticized this protocol on several issues, some of which are listed above [17,19,42]. The trial was presented in Norway as a separate trial, but quite similar studies with the same protocol acronym (ONE) have been reported from Denmark (without ranitidine comparison) [43] and Switzerland [44] in total involving almost 3,500 patients.

## Conclusions

Only one-tenth of all clinical drug trials in Norway involved patients recruited from general practice, and just one in four of these trials were solely general practice trials. Almost all trials were industry initiated without input from academic general practice. This shows that it is a challenge for general practice to increase the number of clinical trials in general and researcher initiated clinical drug trials in particular. Antidiabetic drugs were most commonly studied. There was a large variation in the number of patients, participating doctors, and economic compensation for trial investigators, with some investigators receiving substantial payments. We describe a study with several characteristics of a seeding trial, where none of the official approving bodies for clinical trials had any major remarks. It raises the important ethical considerations with regard to exposing patients to unnecessary risks in either an inordinately large or a small, underpowered trial.

In this descriptive study, we have addressed neither the relevance of the research questions for general practice nor the methodological quality or publication output of the trials beyond the case study. These are important issues that we intend to explore in forthcoming research.

## Abbreviations

ATC code: Anatomical Therapeutic Chemical classification code; DDD: Defined daily dose; ICPC: International Classification of Primary Care; GP: General practitioner; NoMA: Norwegian Medical Agency.

## Competing interests

The authors declare that they have no competing interests. The authors alone are responsible for the content and writing of the paper.

## Authors’ contributions

AMB completed the data collection, analyzed and interpreted the data and drafted the manuscript. KBJ participated in the design of the study, acquired most of the data and critically revised the manuscript. AK analyzed and interpreted the data, and critically revised the manuscript. JS conceived and designed the study, participated in the analysis and interpretation, and critically revised the manuscript. All authors read and approved the final manuscript.
